# Supplementary material for: Iterative improvement in the automatic modular design of robot swarms
Source: PeerJ Comput Sci. 2020 Dec 7;6:e322. doi: 10.7717/peerj-cs.322 (PMC7924708; doi:10.7717/peerj-cs.322)
Supplement: Supplemental Information 3 [file peerj-cs-06-322-s003.zip › argos3/doc/api/standalone/a00468.html]

ARGoS: plugins/robots/foot-bot/simulator/footbot\_light\_rotzonly\_sensor.cpp File Reference


- Main Page
- Related Pages
- Namespaces
- Classes
- Files

- File List
- File Members

# plugins/robots/foot-bot/simulator/footbot\_light\_rotzonly\_sensor.cpp File Reference

`#include <argos3/core/simulator/simulator.h>`  
`#include <argos3/core/simulator/entity/embodied_entity.h>`  
`#include <argos3/core/simulator/entity/composable_entity.h>`  
`#include <argos3/plugins/simulator/entities/light_entity.h>`  
`#include <argos3/plugins/simulator/entities/light_sensor_equipped_entity.h>`  
`#include "footbot_light_rotzonly_sensor.h"`  

Include dependency graph for footbot\_light\_rotzonly\_sensor.cpp:

Go to the source code of this file.

|  |  |
| --- | --- |
| Namespaces | |
| namespace | argos |

|  |  |
| --- | --- |
|  | The namespace containing all the ARGoS related code. |

| Functions | |
|  | argos::REGISTER\_SENSOR (CFootBotLightRotZOnlySensor,"footbot\_light","rot\_z\_only","Carlo Pinciroli [ilpincy@gmail.com]","1.0","The foot-bot light sensor (optimized for 2D).","This sensor accesses a set of light sensors. The sensors all return a value\n""between 0 and 1, where 0 means nothing within range and 1 means the perceived\n""light saturates the sensor. Values between 0 and 1 depend on the distance of\n""the perceived light. Each reading R is calculated with R=(I/x)^2, where x is the\n""distance between a sensor and the light, and I is the reference intensity of the\n""perceived light. The reference intensity corresponds to the minimum distance at\n""which the light saturates a sensor. The reference intensity depends on the\n""individual light, and it is set with the \"intensity\" attribute of the light\n""entity. In case multiple lights are present in the environment, each sensor\n""reading is calculated as the sum of the individual readings due to each light.\n""In other words, light wave interference is not taken into account. In\n""controllers, you must include the ci\_light\_sensor.h header.\n\n""REQUIRED XML CONFIGURATION\n\n"" <controllers>\n"" ...\n"" <my\_controller ...>\n"" ...\n"" <sensors>\n"" ...\n"" <footbot\_light implementation=\"rot\_z\_only\" />\n"" ...\n"" </sensors>\n"" ...\n"" </my\_controller>\n"" ...\n"" </controllers>\n\n""OPTIONAL XML CONFIGURATION\n\n""It is possible to draw the rays shot by the light sensor in the OpenGL\n""visualization. This can be useful for sensor debugging but also to understand\n""what's wrong in your controller. In OpenGL, the rays are drawn in cyan when\n""they are not obstructed and in purple when they are. In case a ray is\n""obstructed, a black dot is drawn where the intersection occurred.\n""To turn this functionality on, add the attribute \"show\_rays\" as in this\n""example:\n\n"" <controllers>\n"" ...\n"" <my\_controller ...>\n"" ...\n"" <sensors>\n"" ...\n"" <footbot\_light implementation=\"rot\_z\_only\"\n"" show\_rays=\"true\" />\n"" ...\n"" </sensors>\n"" ...\n"" </my\_controller>\n"" ...\n"" </controllers>\n\n""It is possible to add uniform noise to the sensors, thus matching the\n""characteristics of a real robot better. This can be done with the attribute\n""\"noise\_level\", whose allowed range is in [-1,1] and is added to the calculated\n""reading. The final sensor reading is always normalized in the [0-1] range.\n\n"" <controllers>\n"" ...\n"" <my\_controller ...>\n"" ...\n"" <sensors>\n"" ...\n"" <footbot\_light implementation=\"rot\_z\_only\"\n"" noise\_level=\"0.1\" />\n"" ...\n"" </sensors>\n"" ...\n"" </my\_controller>\n"" ...\n"" </controllers>\n\n""OPTIONAL XML CONFIGURATION\n\n""None.\n","Usable") |

---

Generated on 10 Jul 2018 for ARGoS by 
 1.6.1
